# Riociguat Reduces Infarct Size and Post-Infarct Heart Failure in Mouse Hearts: Insights from MRI/PET Imaging

**DOI:** 10.1371/journal.pone.0083910

**Published:** 2013-12-31

**Authors:** Carmen Methner, Guido Buonincontri, Chou-Hui Hu, Ana Vujic, Axel Kretschmer, Stephen Sawiak, Adrian Carpenter, Johannes-Peter Stasch, Thomas Krieg

**Affiliations:** 1 Department of Medicine, University of Cambridge, Cambridge, United Kingdom; 2 Wolfson Brain Imaging Centre, University of Cambridge, Cambridge, United Kingdom; 3 Bayer HealthCare, Wuppertal, Germany; Virginia Commonwealth University Medical Center, United States of America

## Abstract

**Aim:**

Stimulation of the nitric oxide (NO) – soluble guanylate (sGC) - protein kinase G (PKG) pathway confers protection against acute ischaemia/reperfusion injury, but more chronic effects in reducing post-myocardial infarction (MI) heart failure are less defined. The aim of this study was to not only determine whether the sGC stimulator riociguat reduces infarct size but also whether it protects against the development of post-MI heart failure.

**Methods and Results:**

Mice were subjected to 30 min ischaemia via ligation of the left main coronary artery to induce MI and either placebo or riociguat (1.2 µmol/l) were given as a bolus 5 min before and 5 min after onset of reperfusion. After 24 hours, both, late gadolinium-enhanced magnetic resonance imaging (LGE-MRI) and ^18^F-FDG-positron emission tomography (PET) were performed to determine infarct size. In the riociguat-treated mice, the resulting infarct size was smaller (8.5±2.5% of total LV mass vs. 21.8%±1.7%. in controls, p = 0.005) and LV systolic function analysed by MRI was better preserved (60.1%±3.4% of preischaemic vs. 44.2%±3.1% in controls, p = 0.005). After 28 days, LV systolic function by echocardiography treated group was still better preserved (63.5%±3.2% vs. 48.2%±2.2% in control, p = 0.004).

**Conclusion:**

Taken together, mice treated acutely at the onset of reperfusion with the sGC stimulator riociguat have smaller infarct size and better long-term preservation of LV systolic function. These findings suggest that sGC stimulation during reperfusion therapy may be a powerful therapeutic treatment strategy for preventing post-MI heart failure.

## Introduction

Chronic heart failure (CHF) is a serious consequence of myocardial infarction (MI) and is associated with high mortality and morbidity. Reducing infarct size acutely after an ischemic event is assumed to reduce the risk of detrimental post-MI remodelling [1] and ensuing CHF. While much effort has gone into the identification of acutely protective strategies to reduce infarct size, relatively little has been directed to actually documenting their long-term post-MI effects.

The NO – sGC - PKG pathway is known to play an important role in the acute protection against cardiac reperfusion injury. We, and others, could show that an increase of cyclic GMP via phosphodiesterase 5 inhibition can afford powerful cardioprotection when applied at the onset of reperfusion. [2], [3] The same is seen with the stimulation of soluble or particular guanylate cyclase. [4], [5] All these interventions seem to require PKG as their downstream target. [6], [7] NO not only can have a trigger role in this pathway at the level of sGC but also appears to be a downstream effecter by directly *S*-nitrosylating protective proteins. [8], [9].

Recently, a new class of drugs, the so-called sGC stimulators, entered the clinical development for the treatment of pulmonary hypertension. Stimulators of sGC have a dual mode of action: they both sensitise sGC to endogenous NO by stabilizing the NO-sGC binding and also directly stimulate sGC independently of NO via a second binding site. The sGC stimulator riociguat, has recently undergone Phase III clinical trials in patients with several subforms of pulmonary arterial hypertension (PAH) and with chronic thromboembolic pulmonary hypertension (CTEPH). Exercise capacity was the primary endpoint for these studies and in all cases riociguat increased the patients' 6-minute-walking-distance. [10]–[12] Additionally, improvements were observed across secondary endpoints, including pulmonary hemodynamics, functional class and time to clinical worsening. Remarkably, riociguat is the first drug that has consistently demonstrated efficacy in both CTEPH and PAH.

Riociguat also produced positive clinical effects in smaller proof-of-concept studies in patients with pulmonary hypertension secondary to left heart failure, interstitial lung disease and chronic obstructive pulmonary disease. [13] In animal models riociguat has been shown to be organ protective against cardiac and renal damage from hypertension and chronic renal failure and volume overload. [13]–[15].

In this study we tested the effects of riociguat on MI size and post-MI CHF development in a mouse model of ischaemia/reperfusion. Gadolinium-enhanced magnetic resonance imaging (MRI) as well as FDG-PET was used to determine the resulting infarct size post-MI, while echocardiogram was used to measure LV function over a 28 day recovery period.

## Methods

All procedures were conducted in accordance with the Animals (Scientific Procedures) Act 1986 (PPL 80/2393) and the University of Cambridge Policy on the Use of Animals in Scientific Research and approved by the Home Office (UK) Animals Scientific Procedures Department (ASPD).

### In vivo mouse model of myocardial infarction

Infarct size following ischaemia/reperfusion an in situ open chest mouse model was measured as previously described. [16] Briefly, male C57/BL6 mice were anaesthetised with either pentobarbital (2 h reperfusion model) or gaseous isoflurane (24 h reperfusion model) and subjected to 30 min occlusion via a snare around the left anterior descending branch of the left coronary artery followed by either 2 h or 24 h reperfusion. Mice received either intravenous saline or 1.2 µmol/l riociguat 5 min before the onset of reperfusion via tail vein injection. L-NAME or KT5823 was given 10 min prior to the riociguat treatment. Cardiac Troponin I was measured in blood serum taken prior to heart removal at the end of each experiment. More details are described in the online supplement.

### Blood pressure measurement

Blood pressure was either measured non-invasively using a tail cuff apparatus or through left ventricular catheterization via the right carotid artery using a 1.2 F pressure-catheter (Scisense Inc., London, Canada).

### Echocardiography assessment of cardiac function

Transthoracic echocardiography was performed on mice anesthetized with 2% isoflurane, using a Vevo 770 ultrasound system (Visual Sonics, Toronto, Canada). Hearts were visualised in the two dimensional short-axis plane and analysis performed in M-mode in the consistent plane of the papillary muscles. Ejection fraction (EF) was calculated from end-systole and end-diastole measurements in at least three repeated cardiac cycles. Fractional shortening was calculated by the Simpson's method.

### MRI and PET imaging

Animals were anaesthetised with gaseous isoflurane both for induction (3% in 1 l/min O_2_) and maintenance (1.5–2% in 1 l/min O_2_). A pressure sensor for respiration rate was used to monitor anaesthesia depth, rate was maintained in the range 30–45 breaths per minute. Prospective gating of the MRI sequences was achieved with ECG monitoring. Body temperature was monitored using a rectal thermometer and a flowing-water heating blanket was used to maintain animal temperature at 37°C throughout the experiment.

MRI was performed at 4.7 T with a Bruker BioSpec 47/40 system (Bruker Inc., Ettlingen, Germany). A birdcage coil of 12 cm was used for signal excitation and a 2 cm surface coil for signal reception with the animals positioned prone. After initial localization images, 4-chamber and 2-chamber views were acquired (FISP, TR/TE 7 ms/2.4 ms, 13–20 frames, 3.5 cm FOV, 256×256 matrix, 1 mm slice thickness, bandwidth 64 kHz, flip angle 20°, NEX2). Using these scans as a reference, short axis slices were arranged perpendicularly to both the long-axis views to cover the left ventricle (LV) (FISP, TR/TE 7 ms/2.4 ms, 13–20 frames, 3.5 cm FOV, 256×256 matrix, 1 mm slice thickness, bandwidth 64 kHz, flip angle 20°, NEX2). Full LV coverage was achieved with no slice gap with 8–10 slices.

DENSE MRI [17] was acquired (3 short-axis slices encoding from end diastole to end-systole, 1 mm thick, TR/TE ∼200 ms/9.5 ms, 3.5 cm FOV, 128 matrix, bandwidth 64 kHz, flip angle 90°, 4 NEX with CANSEL. [18].

After the acquisition of the standard cine protocol and DENSE, late gadolinium enhancement was performed. [19] Contrast agent was injected *in situ* (Gadovist, Bayer; 0.3 mmol/kg i.v.). Within the first 15 min after injection, IR images were acquired (FLASH, FOV 3.5 cm, 256×256, 0.8 mm slice thickness with 0.2 mm gap between adjacent slices TE = 2.8 ms, TR = 550–750 ms, FLASH TR 7 ms, bandwidth 64 kHz, flip angle 60°, 1 NEX, 0.8 mm, 0.2 mm gap, selective inversion 0.8 mm thickness with 5 ms sech shaped pulse).

Animals were transferred on the same bed to the Cambridge PET-MRI scanner. [20] Injection of 25 MBq ^18^F-FDG was performed *in situ*, and list-mode gated PET acquired continuously for 45 min.

### Image Analysis

Delineation of the LV at each phase of the cardiac cycle excluded the papillary muscles and trabeculae throughout. The regions from each slice were combined using Simpson's rule to provide LV mass, end diastolic volume (EDV), end systolic volume (ESV), stroke volume (SV) and ejection fraction (EF) using Segment v1.9. [21] The infarcted regions were manually delineated on the IR images. Values are expressed as ratios of the LV mass as measured from the cine images.

DENSE MRI images were analysed with in-house code using Matlab: phase images were extracted and unwrapped [22] to obtain displacement maps. The Green strain tensor (E) was calculated from the Jacobian matrix (F) by means of E = (F′F-I)/2. The tensor was then decomposed into radial and circumferential strain components. Global strains were obtained by integrating values over the LV.

FDG-PET Images were reconstructed using a 3D filtered backprojection algorithm in 8 cardiac phases and temporal frames of 15 min. The cardiac phase correspondent to end-diastole was selected for each subject. Images from PET and MRI were co-registered manually with a rigid transformation using SPM-Mouse bulk registration tool. [23].

Infarct size was assessed in the FDG-PET images by manual delineation in the final frame. Voxels where intensity was below 50% of the maximum heart uptake were considered non-viable, following Stegger *et al.* [24].

### Relative mRNA quantification by RT-PCR

Details can be found in the online supplement.

### Data analysis

All data are presented as mean ± standard error of the mean (SEM). The infarct size is shown as percentage of area at risk. The blood pressure values are plotted as percentage of the respective baselines. Differences among groups were compared by one-way ANOVA with Turkey's post hoc test. A value of p<0.05 was considered significant.

## Results

### Effects of riociguat on infarct size

**Figure 1:**
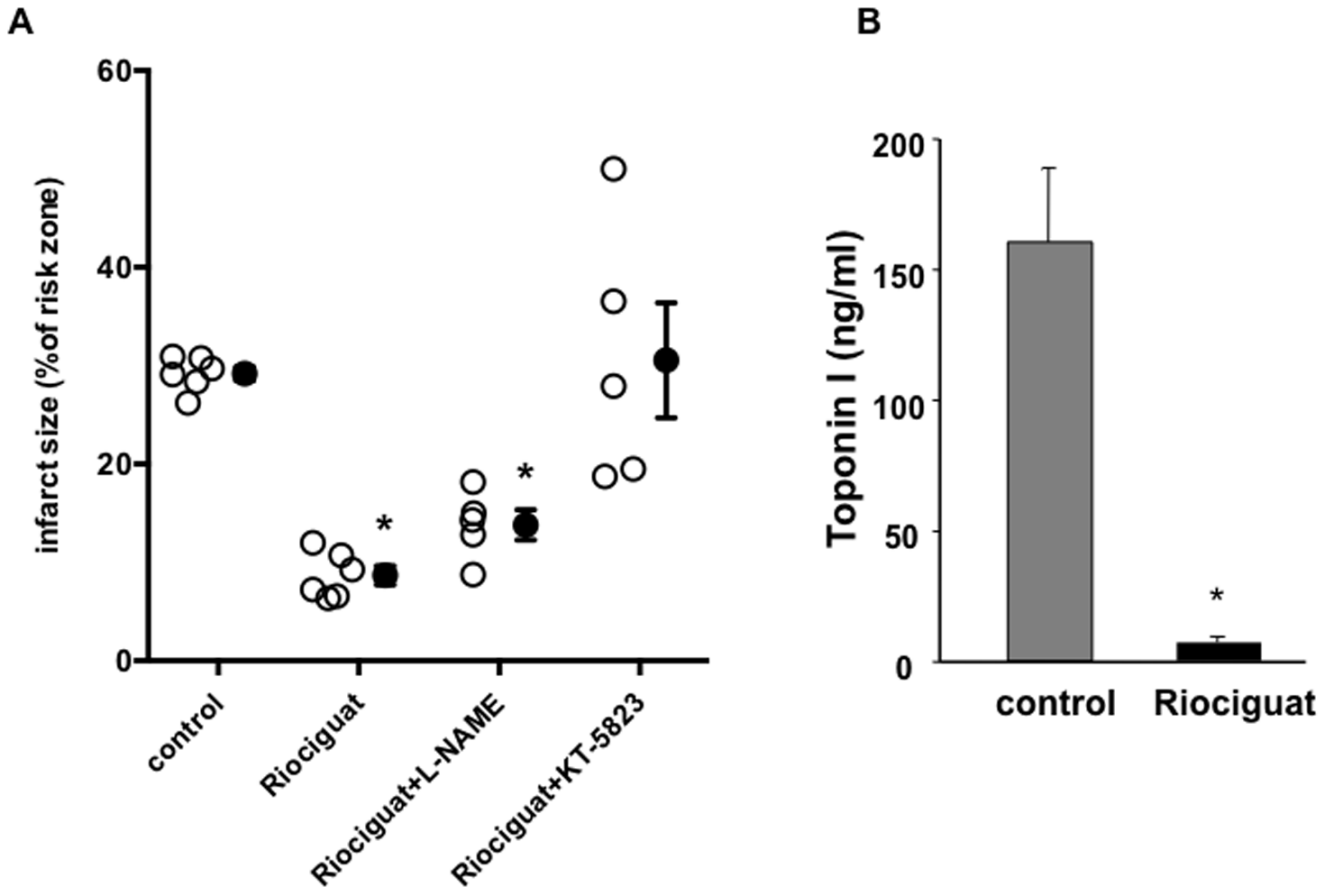
Effects of riociguat on infarct size after acute myocardial infarction. (**A**) Riociguat reduced infarct size significantly when given just before the onset of reperfusion after 30 min ischaemia in an acute *in vivo* mouse model. The protection was unaffected by the NOS inhibitor L-NAME, but blunted when the PKG inhibitor KT5823 was administered prior to riociguat. Open symbols represent individual experiments and closed symbols are means ±SEM. (**B**) Troponin I level in blood serum taken at the end of the 120 min reperfusion further support the results of the histological measurements. *p<0.001.

**Figure 2:**
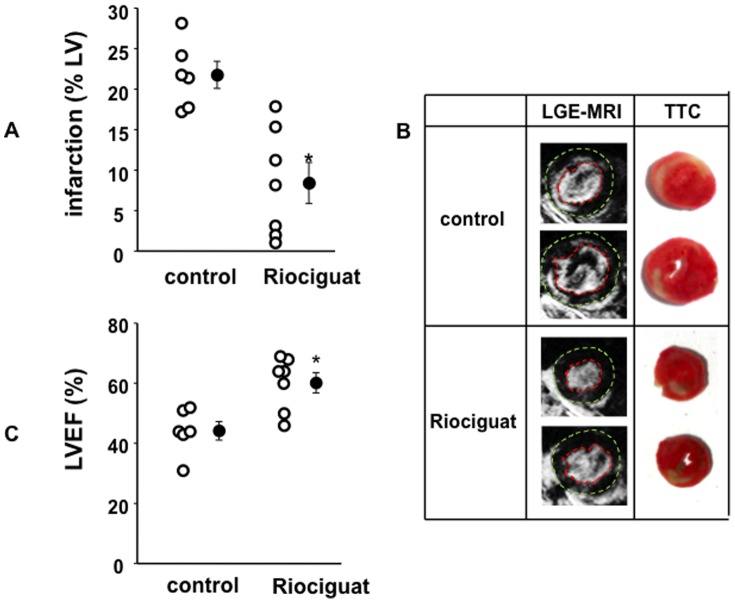
Infarct size assessment with LGE-MRI and effects of riociguat on left ventricular ejection fraction 24 h after myocardial infarction. (A) Infarct size was determined with LGE-MRI 24 h after the ischemic event showing that acute riociguat treatment markedly reduced infarct size. *p<0.005. (B) There was a good correlation between infarct size assessment via histological TTC staining (representative images depictured on the right) and LGE-MRI (left). Epicardial and endocardial borders have been manually added for clarity. (C) Riociguat significantly increased LV ejection fraction compared to untreated control measured by MRI 24 h after the ischemic event. Open symbols represent individual experiment and closed symbols are means ±SEM. *p<0.005.

Mice treated with riociguat at the onset of reperfusion showed a marked reduction of infarct size after 30 min LCA occlusion followed by 2 h reperfusion. The protection was still present when the NOS inhibitor L-NAME was given prior to the riociguat treatment, while the PKG blocker KT5823 blocked the riociguat effect (Fig 1A), suggesting a NO-independent effect through PKG signalling. Infarct size was determined with tetrazolium staining (Fig 1A) and blood serum levels of cardiac Troponin I (Fig 1B). The profound protection affected by riociguat was still present after 24 h when infarct size was determined by late gadolinium enhancement LGE-MRI (Fig 2A, supplemental Table 1 in File S1). Fig 2B shows representative images of LGE-MRI and standard protocol TTC staining for comparison from the same heart after 24 h of infarction. Both techniques for assessment of infarct size show a good correlation in our hands. [19].

**Figure 3:**
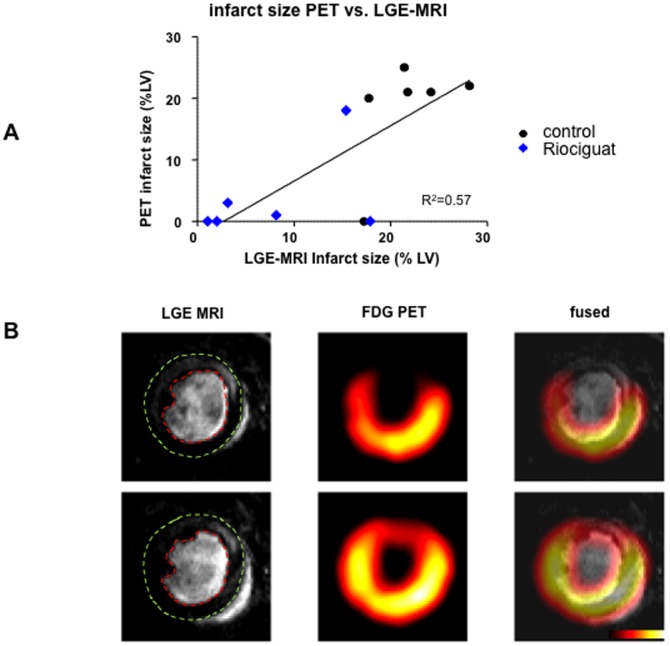
Assessment of infarct size via consecutive LGE-MRI and FDG-PET. (**A**) There was a good correlation between infarct size measured with LGE-MRI and FDG-PET. Acute riociguat treatment at reperfusion (blue) resulted in smaller infarcted areas than untreated controls (black). (**B**) Representative overlay images of LGE-MRI and FDG-PET of a mouse in the control group. Eepicardial and endocardial borders have been manually added on the LGE images for clarity. The infarct area, represented by hyperenhancement in the MRI images, matches an area of hypoenhancement on the PET.

Furthermore, FDG-PET was used as an additional technique to assess functional infarct size data. As shown in Figure 3A, the direct comparison of infarct size measurements between LGE-MRI and PET reveal a good correlation between these two techniques. Fig 3B depicts example images of LGE-MGI and PET as well as the overlay of both techniques. Representative films of merged LGE-MRI/PET can be seen for control and riociguat-treated mice in the online supplement (Videos S1 and S2).

### Effects of riociguat on cardiac function and heart failure development

**Figure 4:**
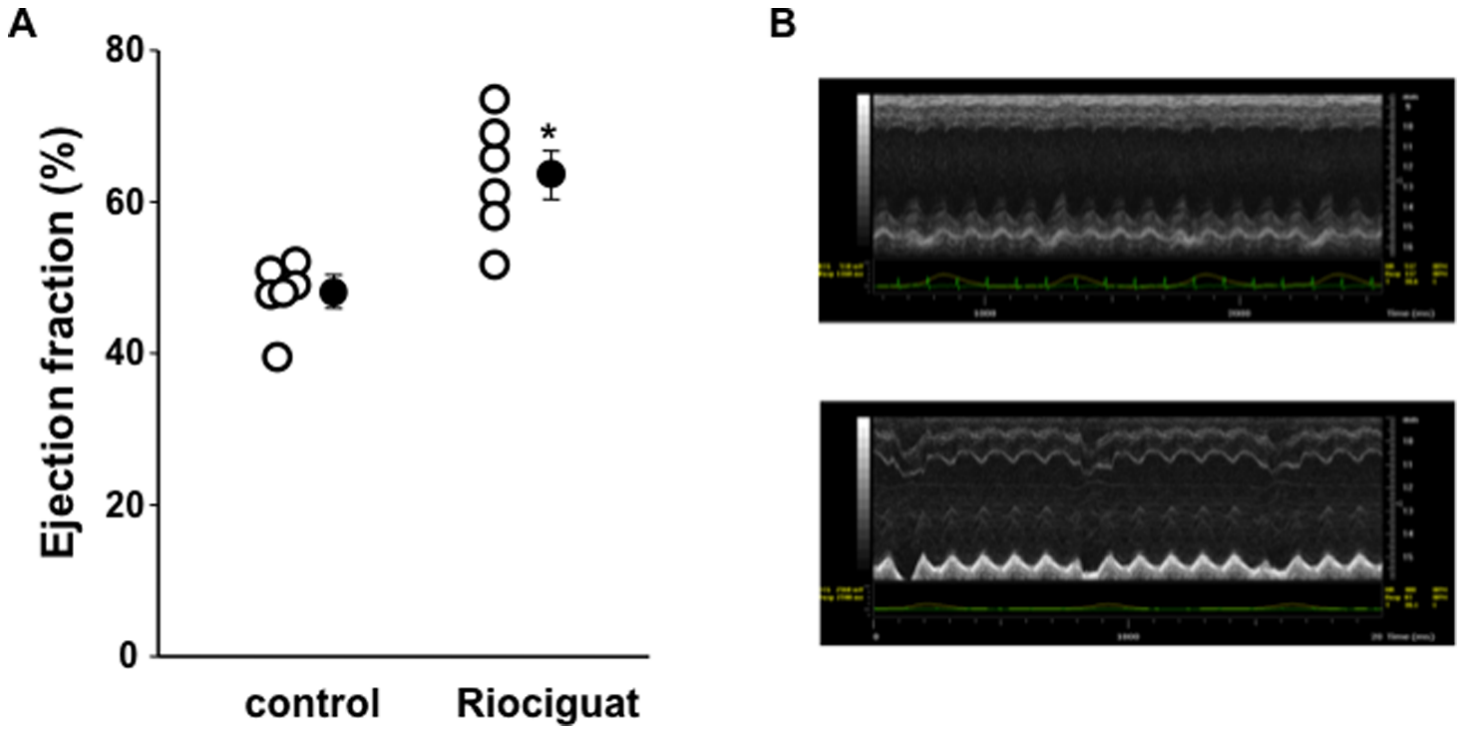
Improvement of LV ejection fraction following riociguat treatment 28 days post-MI. (**A**) Early riociguat treatment continued to result in improved LV ejection fraction, suggesting beneficial effects on post-MI remodelling. *p<0.004. (**B**) Representative images of M-mode traces of control (top) and riociguat treated (bottom) mice hearts.

Left ventricular ejection fraction (LVEF) was assessed with cardiac MRI 24 h post-MI and with ultrasound 28 days later (for a technical comparison of both techniques see supplemental Fig 1 and 2 in File S1). LVEF in the riociguat-treated mice was much greater compared to control at this early time-point (Fig 2C). After 28 days, echocardiography still revealed a markedly greater LVEF in the riociguat treated mice (Fig 4, supplemental Table 2 in File S1), suggesting a long-lasting beneficial effect of a single dose of riociguat administered at the time of reperfusion. The preserved LVEF at 28 days was highly correlated with infarct size 24 h post MI in the same animals (R^2^ = 0.85, supplemental Fig 2 in File S1).

**Figure 5:**
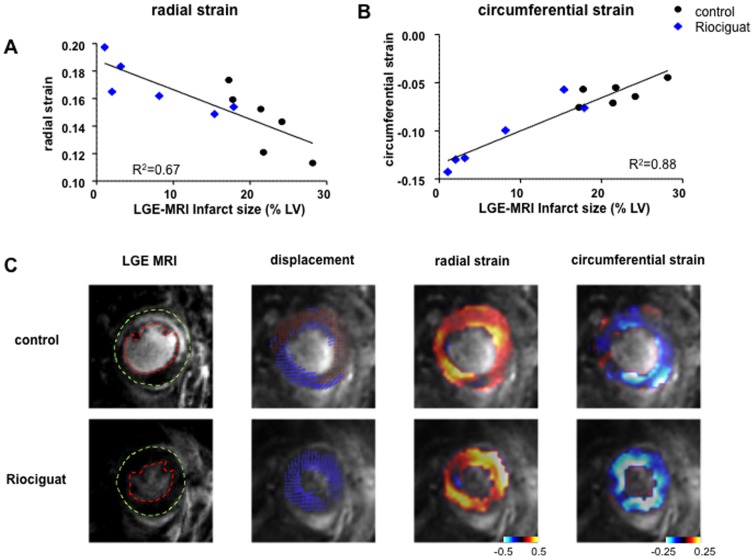
Assessment of cardiac deformation after myocardial infarction. Radial (**A**) and circumferential (**B**) strain as representative markers of heart deformation were obtained 24 h after infarct with LGE-MRI Both parameter show marked improvement in heart function after treatment with riociguat (blue) compared to untreated control (black). Representative images are also shown for each of the parameters. Endocardial and epicardial borders have been delineated on the LGE images, hypokinetic areas have been marked in red in the displacement images.

MRI also allowed us to measure the ventricular wall's radial and circumferential strains one day post-MI. Radial strain is an indicator of the change in wall thickness during contraction. Hearts treated with riociguat had greater radial strain, indicating more wall thickening during systole when compared to the control group (Fig 5A). Circumferential strain reflecting circumferential shortening with systole and healthy myocytes should have negative values. Fig 5B shows a clear improvement in both strains for hearts treated with riociguat.

### Effects of riociguat on hemodynamics

**Figure 6:**
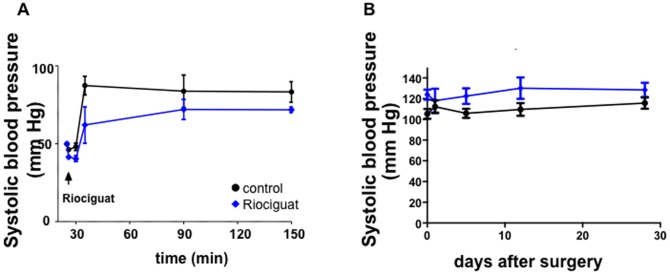
Effects of riociguat on hemodynamics. (**A**) Systolic blood pressure data were obtained by using a LV-catheter during infarct size measurement in the acute model with 30 min ischaemia followed by 2 h reperfusion. There was a slight blood pressure drop after riociguat injection compared to the vehicle-treated mice in the control group, although changes are not significant. n = 3. (**B**) Long term systolic blood pressure data were obtained by using a tail cuff system. One day after injection of riociguat systolic blood pressure still showed a mild trend towards lower values, which fully disappeared later. n = 7.

Blood pressure measurements during the open-chest phase of myocardial infarction via an LV catheter showed a mild drop and a slightly slower recovery in mean blood pressure after the infusion of riociguat (Fig 6A). These differences did not reach significance, however. While this trend was still seen at 24 h post-MI, it was not seen at any of the later stages between the groups (Fig 6B). Heart rate was not significantly different in the control animals compared to riociguat treated mice. (Supplemental Fig 3 in File S1).

### Effects of riociguat on cardiac fibrotic tissue remodelling

**Figure 7:**
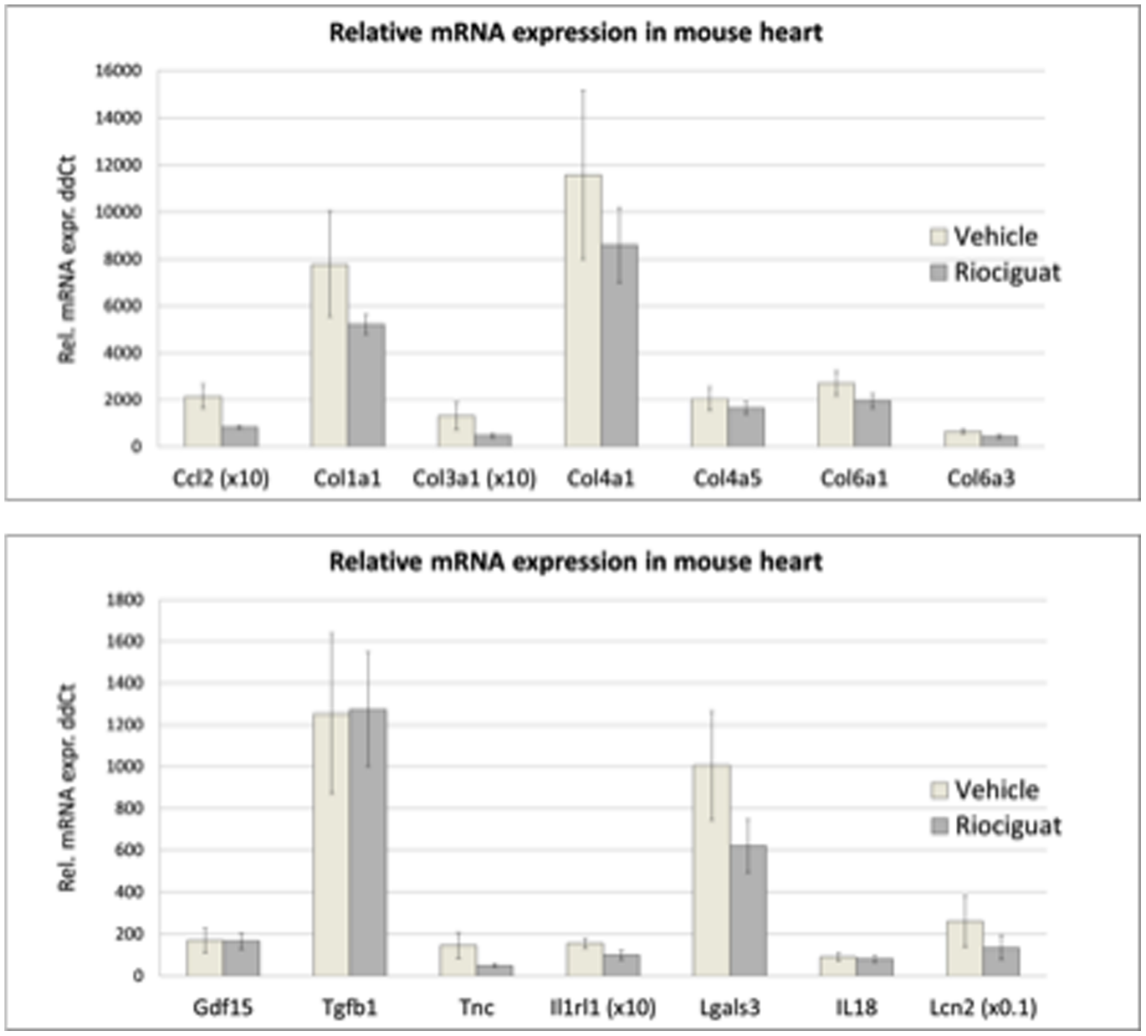
Relative mRNA expression in myocardium of vehicle or riociguat treated mice at day 28 post MI. Riociguat treatment resulted in a trend towards reduced levels of pro-fibrotic gene expression in MCP-1 (Ccl2), tenascin C, ST2 (Il1rl1), galectin-3 or lipocalin-2. Due to the high variation, significance was not reached for any of the genes tested gene. n = 7.

After sacrifice at 28 days, mRNA gene expression profiles in left ventricles of the mice were quantified by RT-PCR in order to characterize tissue remodelling processes. Although not significant, the average expression of Collagenes (Col1a1, Col3a1, Col4a1, Col6a1) were slightly less in myocardium of riociguat-treated mice compared to that in the untreated controls. This may indicate an attenuated fibrotic tissue remodelling process in the ventricle, when sGC activity is stimulated at the onset of myocardial reperfusion. Consistent with reduced expression of extracellular matrix molecules after riociguat treatment, other pro-fibrotic gene activities also tended to be expressed at a lesser extent, i.e. MCP-1 (Ccl2), tenascin C, ST2 (Il1rl1), galectin-3 or lipocalin-2 (Fig 7). Unfortunately none of the values in Fig 7 reached significance.

## Discussion

In this study we present clear evidence for a strong and long-lasting cardioprotective effect of riociguat, a novel sGC stimulator in an *in vivo* mouse model of MI and post-MI CHF. Riociguat not only acutely reduced infarct size and improved LV function but the effect persisted over the 28 days of the study. Infarct size was not only evaluated histologically with TTC staining, but also with novel state-of-the-art imaging techniques. LGE-MRI and FDG-PET were used 24 h after reperfusion and revealed beneficial effects of sGC stimulation on infarct size, cardiac function and morphology. The follow-up over 28 days showed a sustained preservation of mechanical function from the single riociguat treatment at reperfusion indicating that the entire benefit was derived from preventing acute loss of ventricular muscle.

NO activates PKG by causing SGC to increase cGMP in the cell. Impairments of the NO-sGC-PKG pathway have been implicated in various cardiac pathologies, including ischaemia/reperfusion injury and CHF development. [5] Riociguat is a potent sGC stimulator, which is currently being investigated in phase III clinical trials for the treatment of pulmonary hypertension. Riociguat acts directly on sGC and sensitizes sGC to endogenous NO. [13], [25] In animal models of hypotension riociguat afforded protection against cardiac and renal end organ damage [15] and it showed reduced left ventricular weight and cardiac interstitial fibrosis in low and high renin rats. Furthermore, in a model of chronic cardiac volume and pressure overload in salt sensitive rats riociguat reduced systemic hypertension and cardiac fibrosis as well as increased systolic heart function and survival. [14], [15] In the present study, we could observe a non-significant, non-sustainable trend towards a mild reduction in systolic blood pressure in the riociguat-treated animals. We cannot rule out any effect of the blood pressure changes on infarct size or CHF development, although it is known that blood pressure reduction *per se* does not afford cardioprotection against ischaemia-reperfusion injury [26], [27].

In recent years, imaging techniques such as MRI and PET became available for the functional and morphological assessment of mouse hearts and allowed non-invasive follow-up studies of MI and CHF. Here we used state-of-the-art techniques to assess myocardial function and morphology with the consecutive combination of LGE-MRI and FDG-PET. While the cardiac MRI provides accurate functional data on the basis of detailed morphology, PET is a highly specific complimentary technique providing functional data on a molecular level. The combination of both techniques overcomes the clear shortcomings of each one alone and represents the current gold standard in *in vivo* cardiac imaging. When we used LGE-MRI to assess infarct size after 24 h of reperfusion there was still a clear benefit in the riociguat-treated animals with smaller infarct sizes and better-preserved LV function. All the techniques used were highly congruent.

Furthermore, DENSE MRI imaging allowed us to determine displacement maps and calculate radial and circumferential strain. [17], [22] Both parameters showed a clear benefit of early riociguat-treatment post-MI with an increase in radial strain, suggesting thicker cardiac walls, and a reduced circumferential strain, indicating preserved myocyte contraction. Global values from tissue deformation imaging have shown high prognostic value for remodelling in infarct patients, and it has been suggested that these might more closely reflect myocardial contractility than traditional measures of systolic function [28].

These non-invasive imaging techniques allowed us to perform a follow-up of these animals and determine functional parameters after 28 days via echocardiography. Human data suggests that remodelling and CHF development is directly correlated to the extent of the infarcted area after an acute MI. Here we could show that the infarct size reduction due to early riociguat treatment was associated with improved LV function after 4 weeks, suggesting less detrimental post-MI remodelling. This is supported by our observation that many pro-fibrotic genes show a strong trend for lower expression in the treated animals' hearts.

Taken together, the present results indicate that a single dose of the sGC stimulator riociguat at the end of a 30 min period of coronary arterial branch occlusion cause an immediate reduction of infarct size and preservation of cardiac function in mice. Furthermore, the beneficial effects on cardiac function and morphology can still be seen 28 days after the ischemic event, leading to a reduction of CHF. The consecutive combination of cardiac LGE-MRI and FDG-PET allowed us to accurately assess infarct size non-invasively in a myocardial infarction model that is very close to the clinical scenario. We conclude that sGC stimulation with riociguat is a promising candidate for preventing post-MI heart failure in acute coronary syndrome patients undergoing reperfusion therapy.

## Supporting Information

File S1 **Supplemental methods and results.**
(DOCX)

Video S1 **Merged LGE-MRI/PET video 24 h after infarction of representative heart of control-treated mouse in 2-chamber view.**
(WMV)

Video S2 **Merged LGE-MRI/PET video 24 h after infarction of representative heart of riociguat-treated mouse in 2-chamber view.**
(WMV)
