# Supplementary material for: Riociguat Reduces Infarct Size and Post-Infarct Heart Failure in Mouse Hearts: Insights from MRI/PET Imaging
Source: PLoS One. 2013 Dec 31;8(12):e83910. doi: 10.1371/journal.pone.0083910 (PMC3877128; doi:10.1371/journal.pone.0083910)
Supplement: File S1 — Supplemental methods and results. (DOCX) [file pone.0083910.s001.docx]

**Supplementary methods**

***In vivo mouse model of myocardial infarction***

For the 2 h reperfusion infarct size model the myocardial infarction was induced in mice as previously described. [1] All animals were subjected to 30 min occlusion and 2 h reperfusion of the left anterior descending branch of the left coronary artery. Mice received either intravenous saline or 1.2 μmol/l riociguat 5 min before the onset of reperfusion via tail vein injection. L-NAME (2 mg/kg) or KT5823 (1 mg/kg) was given intraperitoneal 10 min before the riociguat administration. Cardiac Troponin I was measured in blood serum taken prior to heart removal at the end of each experiment.

For the 28 day reperfusion model mice underwent myocardial infarction as followed. Male mice (age 8-10 weeks) were anaesthetized with gaseous isoflurane, intubated endotracheally, and ventilated with 3 cm H_2_O positive end-expiratory pressure and ventilation frequency of 240 bpm. A small thoracotomy was performed, and the heart was exposed by means of stripping of the pericardium. The main branch of the left coronary artery (LCA) was identified and ligated for 30 min using a 7.0 suture. The thoracic incision was closed and mice were monitored until recovery. Buprenorphine (0.05 mg/kg) was given subcutaneously just prior the end of the surgery and during the recovery period as necessary. All mice underwent LGE-MRI after 24 h of infarction and echocardiography after 28 days.

***Comparison of MRI and ECHO in LVEF assessment***

To compare MRI and echocardiogram techniques in mice, experiments were performed utilising *n=5* wild type mice and *n=5* heart failure mice (4 weeks after ischaemic injury). A Bland-Altman plot was used to assess agreement between ECHO and MRI. Results, reported in Fig 3, show an agreement not dissimilar from previously reported studies [2]. In our hands ECHO tends to overestimate MRI by about 5%.

***Relative mRNA quantification by RT-PCR***

Left ventricles were taken directly after sacrificing the mice and immediately frozen on dry ice and stored at – 80^o^C in order to isolate total RNA. Frozen tissue samples were ground in liquid nitrogen and extracted in 900 µl TRIzol according to the protocol provided by Invitrogen Corp. using glass bead homogenization in a FastPrep FP120 Bio-101 Savant vibration mill. Subsequently to phenol and chloroform extraction and precipitation by isopropanol, DNA was eliminated from RNA samples by deoxyribonuclease I digest (Invitrogen). The ImProm-II TM Kit, (Promega) was used for reverse transcription and first-strand cDNA synthesis.

Relative gene expression was determined by real-time quantitative polymerase chain reaction using the ABI Prism Sequence Detection System (Applied Biosystems ABI Prism 7700 Sequence Detector). cDNA samples were amplified with a PCR mix containing Taq polymerase (qPCR MasterMix Plus, Eurogentec, Belgium) and primer sets with 6-FAM and TAMRA labelled probes (Operon Biotechnologies, Cologne, Germany) in 384-well microtiter plates. 1-10ng of cDNA samples were run in triplicates in reaction volumes of 20 µl under standard thermocycler conditions.

TaqMan probe sets were generated from mRNA sequence data (NCBI Genbank, U.S. National Library of Medicine, Bethesda, MD) using Primer Express Software v2.0 (Applied Biosystems). Relative gene expression was calculated using the delta Ct term (Applied Biosystems) related to endogenous controls ribosomal protein L32 and beta-actin. The primer probe sets were generated from the following sequences:

**Supplementary tables and figures**

|  | **control** | **riociguat** |
| --- | --- | --- |
| **LVM (μl)** | 99 ± 4 | 92 ± 5 |
| **LVEDV (μl)** | 56 ± 3 | 46 ± 3* |
| **LVESV (μl)** | 32 ± 3 | 18 ± 1** |
| **LVSV (μl)** | 24 ± 1 | 27 ± 3 |
| **LVEF (%)** | 44 ± 3 | 60 ± 4** |
| **RVEDV (μl)** | 36 ± 2 | 41 ± 4 |
| **RVESV (μl)** | 13 ± 2 | 14 ± 1 |
| **RVSV (μl)** | 24 ± 1 | 27 ± 3 |
| **RVEF (%)** | 66 ± 3 | 66 ± 1 |
| **MRI - Infarct size (%LV)** | 22 ± 2 | 8 ± 3** |
| **Radial strain** | 0.144 ± 0.009 | 0.168 ± 0.007* |
| **Circumferential strain** | -0.061 ± 0.004 | -0.106 ± 0.013* |
| **PET - Infarct size (%LV)** | 18 ± 4 | 4 ± 3** |

**Supplemental Table S1: Key values obtained by MRI 24 h after myocardial infarction from control and riociguat treated mice.** LVM Left ventricular mass, LVEDV Left Ventricular End-Diastolic Volume, LVESV Left Ventricular End-Systolic Volume, LVSV Left Ventricular Stroke Volume, LVEF Left Ventricular Ejection Fraction, RVEDV Right Ventricular End-Diastolic Volume, RVESV Right Ventricular End-Systolic Volume, RVEV Right Ventricular Stroke Volume, RVEF Right Ventricular Ejection Fraction. Values are mean ± sem. **p*<0.05 ***p*<0.01

|  | **control** | **riociguat** |
| --- | --- | --- |
| **IVS d** | 0.927 ± 0.04 | 0.776 ± 0.05 |
| **LVID d** | 4.229 ± 0.18 | 4.363 ± 0.22 |
| **LVPW d** | 0.911 ± 0.10 | 0.867 ± 0.06 |
| **IVS s** | 1.327 ± 0.05 | 1.166 ± 0.08 |
| **LVID s** | 3.801 ± 0.50 | 2.955 ± 0.24 |
| **LVPW s** | 1.038 ± 0.09 | 1.363 ± 0.07* |
| **LVEF (%)** | 48.2 ± 2.2 | 63.5 ± 3.2** |
| **HR** | 516 ± 32 | 527 ± 31 |

**Supplemental Table S2: Key values obtained by Echo after 28 days of infarction from control and riociguat treated mice.** IVS Intraventricular septal width, LVID left ventricle internal diameter and LVPW left ventricle posterior wall were measured during diastole (d) and systole (s). HR heart rate, LVEF left ventricular ejection fraction. Values are mean ± sem. **p*<0.05 ***p*<0.01


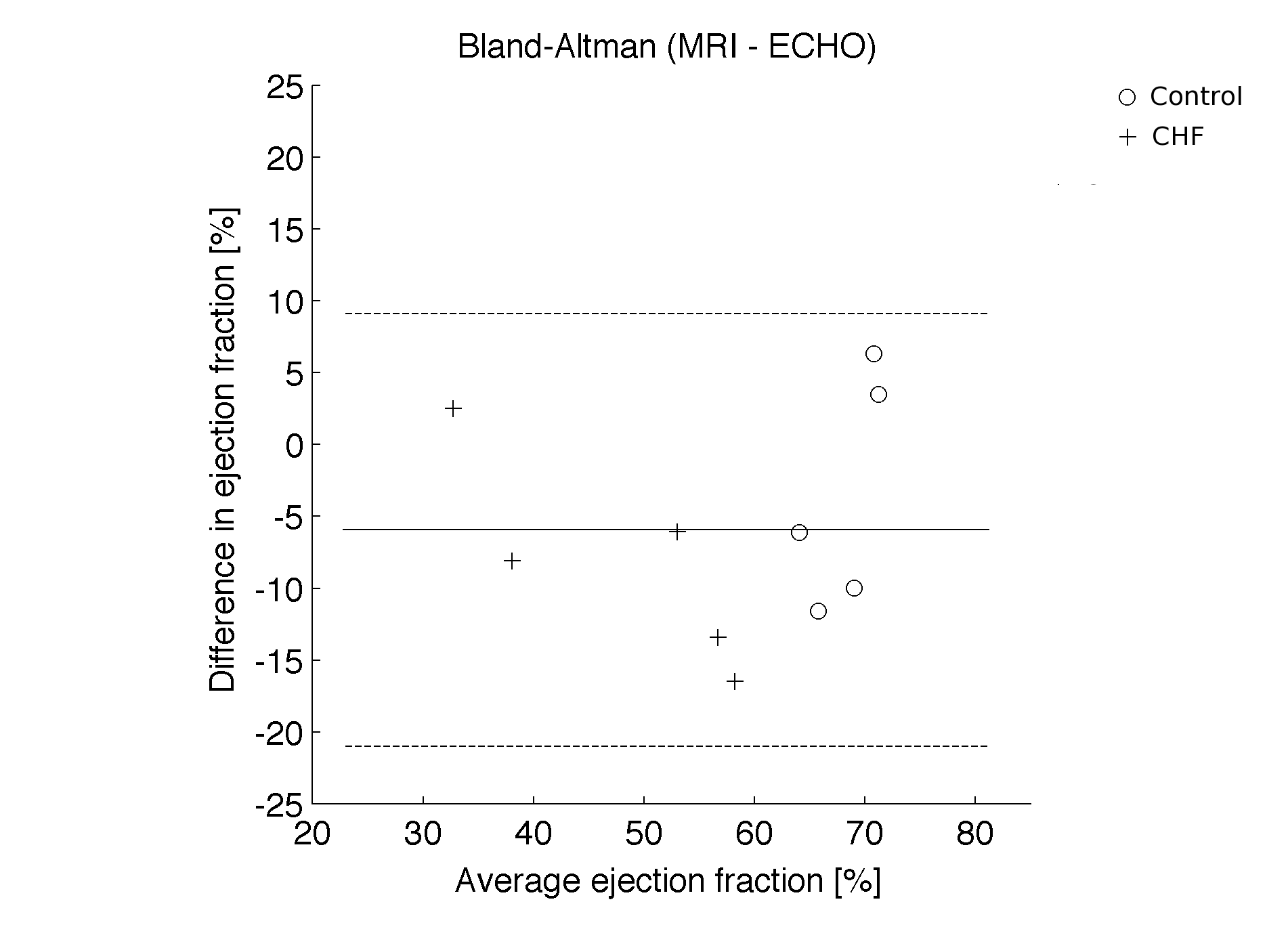


**Supplemental Fig S1: Comparison between MRI and ECHO assessment of LVEF.** Bland-Altman plot showing a high degree of agreement between LVEF assessment with echocardiogram and cine MRI.


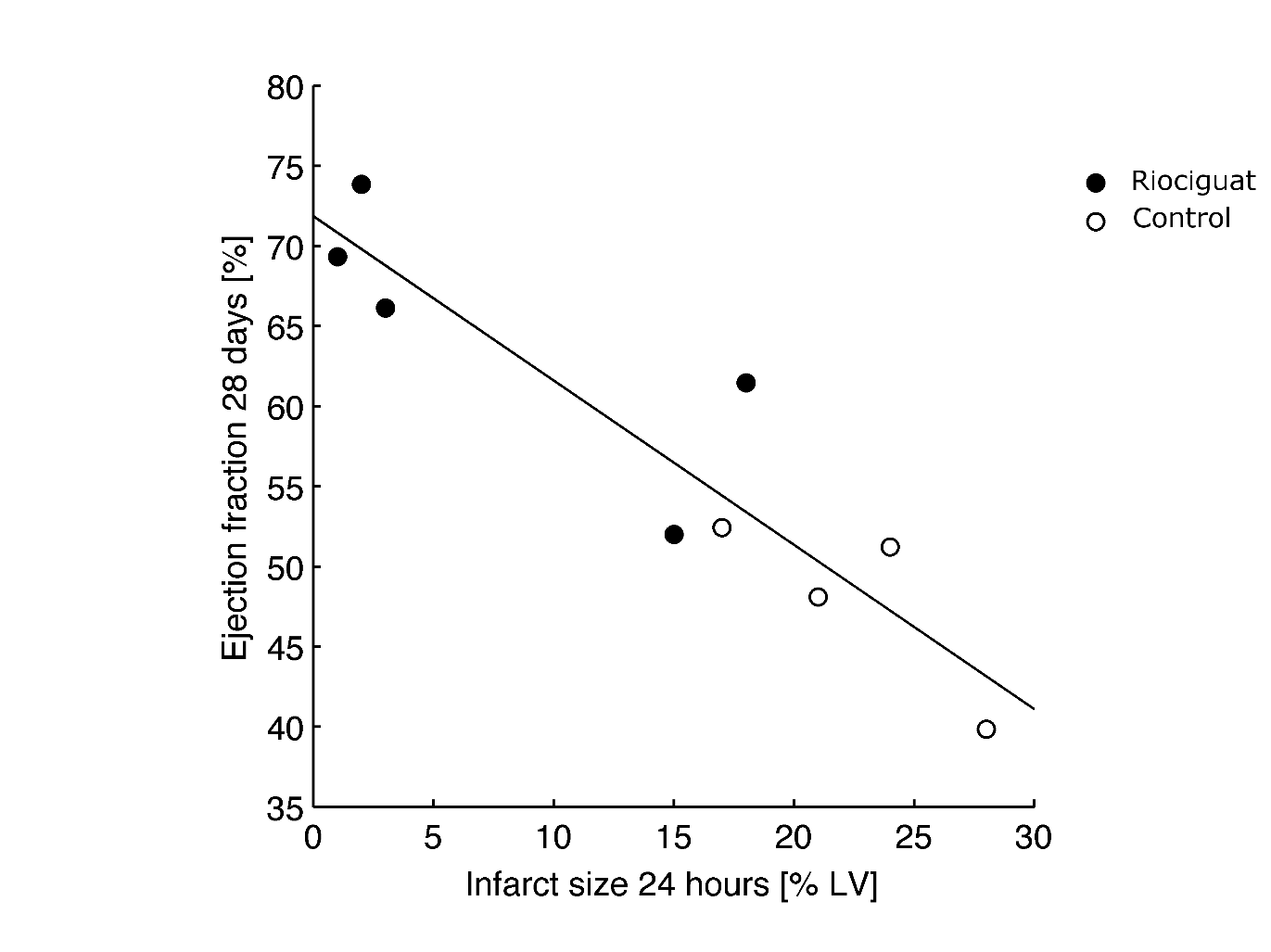


**Supplemental Fig S2: Correlation between infarct size at 24 hours measured with LGE MRI and ejection fraction at 28 days measured with ECHO in the same mice**. The high correlation (R^2^=0.85) indicates that most of the positive outcome at 28 days is predicted by the infarct size at 24 hours.

A B

**Supplemental Fig S3: Effects of riociguat on heart rate.** Heart rate measurements at the acute stage showed a slightly not significant drop in heart rate in the control animals compared to the riociguat treated animals (A). This difference was vanished after 24 h of surgery (B).

**Supplemental videos:**

Merged LGE-MRI/PET video 24h after infarction of either representative heart of the control (Supplemental Video S1: Control2ch.wmv) or riociguat-treated mice (Supplemental Video S2: Riociguat2ch.wmv) in 2-chamber view.

**Supplement Reference:**

1. Methner C, Schmidt K, Cohen M V, Downey JM, Krieg T. (2010) Both A_2a_ and A_2b_ adenosine receptors at reperfusion are necessary to reduce infarct size in mouse hearts. Am J Physiol Heart Circ Physiol 299: H1262–4.

2. Stuckey DJ, Carr CA, Tyler DJ, Clarke K. (2008) Cine-MRI versus two-dimensional echocardiography to measure in vivo left ventricular function in rat heart. NMR Biomed 21: 765-772.
